# Bleaching Discolored Teeth

**Published:** 1867-06

**Authors:** J. H. M'Quillen


### SELECTED ARTICLES.

### ARTICLE VIII.

### *Bleaching Discolored Teeth.*

By Professor J. H. M'Quillen, M.D., D.D.S.

It is a somewhat remarkable and inexplicable fact that none of the text-books which have been presented to the profession, so far as my observation goes, pay even the compliment of a passing notice to the means whereby discolored teeth may be improved in appearance. Whether the subject was regarded as unimportant—the methods so well known as not to require description or comment—or the authors did not know of any other means of eradicating such difficulties other than by extracting the offending organs, is hard to say; and yet when recalling instances in which the beauty and symmetry of sets of teeth that truly rivaled pearls in their color, brilliancy and perfection of structure and form, have been entirely marred by an unsightly blackened or discolored tooth, the importance of the subject becomes apparent, and it is not surprising that the presence of such teeth should be a constant source of mortification and annoyance to the patients, or that they should desire to be relieved in some way or other from the source of trouble. When all efforts to remove the discoloration has proved unavailing, a strongly developed feeling of pride of appearance has not unfrequently induced patients to insist upon extraction, and practitioners have been found willing to comply with the request, although in all other respects the teeth were good and useful organs.

The shades of color presented by such teeth vary greatly, and depend upon the cause producing the discoloration and length of time that it has existed ; thus it may be of a rosy or even a scarlet hue when recent ; or brown, greenish, or black when of long-continued duration. A fall, a blow, or exposure to thermal influences may do such violence to a tooth that the vessels of the pulp will become congested to an extent that rupture of the blood corpuscles will take place, and the hæmotine or coloring matter of the blood uniting with the liquor sanguinis, being carried into the dentinal tubuli, gives to the tooth a rosy appearance, and in the case of very young persons, where the dentinal tubuli are very large, the color sometimes reaches a brighter red. The treatment indicated under such circumstances is to drill at once into the pulp cavity, so as to afford a convenient place of exit for the blood. In some cases the discoloration will disappear in a very short time after the removal of the pulp by syringing the pulp cavity with tepid water ; in other instances the employment of additional agencies, which will he named hereafter, may be required.

Devitalization of the dental pulp may ensue from the causes referred to above, with little or no evidence of the fact until the attention of the patient or friends is arrested by a slightly darkened appearance of the tooth. It is in cases such as this that the acuteness of vision and judgment of the dentist are frequently put to the test, and it is important that he should be prepared to decide promptly, so as to afford the proper attention immédiately, and prevent additional and permanent discoloration. In cases of doubt, the employment of a strong, clear, and steady light, falling directly upon the tooth, combined with the reflection from a good mouth mirror held back of it, will afford an opportunity to institute a comparison between the affected organ and the adjacent healthy ones, and readily decide the question. In other cases, the extreme dark discoloration tells the tale in a forcible manner. In the treatment of exposed pulps it is not an unusual thing, in the hands of careless practitioners, for the teeth treated to become discolored, and even with the greatest care on the part of the experienced, skillful, and pains-taking, some evidence of the loss of vitality will occasionally be made manifest by a slight change of color in teeth treated by them.

The coloring matter of the blood absorbed by the tubuli remaining there, changes to a dark or blackish hue, and imparts to the tooth the variety of shades already referred to, or the absorbtion of the oral secretions, mixed with foreign substances, in teeth where the pulp cavities are allowed to remain open for some time, may induce the same result. Too much care, indeed, cannot be exercised in protecting a tooth from such influences.

As in the case of the rosy tooth, the first indication when treating a dark discolored tooth, is to open into the pulp cavity, if not already exposed, with a drill; or remove the filling in a case where the pulp has been treated with the arsenical paste, and then syringe the cavity freely with water, so as to remove all decomposed or foreign substances; after this the employment of one or the other of the following combinations of chlorine with soda, lime, or potash, will be found, as a general thing, efficacious in restoring a tooth to its natural color. The active agent in bringing about this result, of course, is the chlorine, whose chief characteristic is the bleaching power it possesses; decomposing in a rapid and remarkable manner the most stable organic coloring principles, by combining with and removing the hydrogen present in the coloring matter. On account of this property it is largely used in the arts, particularly in bleaching linen and cotton goods prior to their employment in the manufacture of paper.

Labarraque's liquid, the Liquor Sodæ Chlorinatæ, U. S. Dispens., is one of the most reliable articles in bleaching discolored teeth, and much less objectionable than the other preparations, which will be named. When using it, a pledget of cotton of suitable size, to admit of an easy passage into the bulbous portion of the pulp cavity, should be saturated with the liquid and placed in the tooth, and allowed to remain there about thirty minutes or so; during this time the fluid permeating the dentinal tubuli comes in contact with the coloring matter and decomposes it. In teeth slightly discolored, a single application may suffice, while repeated applications will be demanded in cases where the discoloration is very great or long continued. Care should be exercised in making the application not to allow it to cause unnecessary annoyance by coming in contact with the tongue, as the taste is very disagreeable. This can be readily prevented by covering the pledget of cotton with a temporary stopping of wax and cotton.

Chloride of Lime, or Calx Chlorinata, U. S. Disp., the bleaching powder of commerce, is sometimes employed by dental practitioners, and when used with care is a very valuable agent; but its exceedingly disagreeable odor, and its powerfully destructive action on organic structures, are objections which demand that it should be employed with the greatest caution, or it may do more harm than good. This article when fresh and well prepared is a soft, white powder, which attracts moisture from the atmosphere, and is soluble in about ten parts of water. In this connection it may be well to state that the presence of water is essential in securing the bleaching properties of chlorine, for the gas, in a state of perfect dryness, is incapable even of effecting litmus paper. It should be applied on a pledget of cotton in the same manner as the article first named.

Chlorate of Potash, Potassæ Chloras, Lond. Pharm., may be employed with advantage, but, like the chloride of lime, must be used with judgment and discretion; either of these articles, in the hands of ignorant or careless operators, may become a source of great discomfort to patients by exciting intense irritation and subsequent inflammation in the perodental membranes. The proper way to prevent this is to fill the cavity in the roots with cotton prior to making the application.

In the intervals between the application of the chlorinated preparations, the pulp cavities should be protected from external influences as much as possible by temporary but efficient stoppings.—*Dental Cosmos.*

