# Correction

**Published:** 1867-07

**Authors:** 


*Correction.*—In the June No. of *Dental Cosmos* appears a very favorable notice of the *Am. Jour. of Dental Science*, for which we feel much indebted to Prof. McQuillen.

In the same article reference is made to a paragraph which appeared in the Editorial matter of the May No. of our Journal, confessing to a feeling of disappointment on our part that the *Cosmos* had passed over without notice, certain minutes in the proceedings of the Association of the Colleges of Dentistry. We cheerfully take the first opportunity presenting, to correct the paragraph referred to, the able article of Prof. McQuillen on Dental Education, published in the May No. of the *Cosmos*, rendering this acknowledgment necessary. It is but doing justice to the Editor of the *Cosmos* to say, that he was very decided and uncompromising in his opposition to the conferring of degrees irregularly, while the subject was being discussed at the last meeting of the Association.

